# Recent Progress and Future Prospective in HBV Cure by CRISPR/Cas

**DOI:** 10.3390/v14010004

**Published:** 2021-12-21

**Authors:** Yu-Chan Yang, Hung-Chih Yang

**Affiliations:** 1 Department of Microbiology, College of Medicine, National Taiwan University, Taipei 10051, Taiwan; d00445003@ntu.edu.tw; 2 Graduate Institute of Clinical Medicine, College of Medicine, National Taiwan University, Taipei 10051, Taiwan; 3 Department of Internal Medicine, National Taiwan University Hospital, Taipei 10002, Taiwan

**Keywords:** hepatitis B virus (HBV), chronic hepatitis B (CHB), HBV cure, CRISPR-Cas9, gene editing

## Abstract

Hepatitis B virus (HBV) infection remains an important issue of global public health. Although current antiviral therapy has dramatically reduced the mortality and morbidity of chronic hepatitis B (CHB), it fails to cure it. Rebound viremia often occurs after stopping antiviral therapy. Persistent HBV covalently closed circular DNA (cccDNA) and integrated DNA under antiviral therapy form the major barrier to eradication of HBV infection. CRISPR-mediated genome editing has emerged as a promising therapeutic approach to specifically destroy persistent HBV genomes, both cccDNA and integrated DNA, for HBV cure. However, the cleavage of integrated HBV DNA by CRISPR-Cas9 will cause double-strand break (DSB) of host genome, raising a serious safety concern about genome instability and carcinogenesis. The newly developed CRISPR-derived base editors (BEs), which fuse a catalytically disabled nuclease with a nucleobase deaminase enzyme, can be used to permanently inactivate HBV genome by introducing irreversible point mutations for generation of premature stop codons without DSBs of host genome. Although promising, CRISPR-mediated base editing still faces daunting challenges before its clinical application, including the base-editing efficacy, the off-target effect, the difficulty in finding conserved target HBV sequences, and in vivo delivery efficiency. Several strategies have been adopted to optimize the efficiency and specificity of CRISPR-BEs and to improve in vivo delivery efficacy through novel viral and non-viral delivery approaches. Particularly, the non-viral delivery of Cas9 mRNA and ribonucleoprotein by lipid nanoparticles exhibits attractive potential for liver-targeted delivery in clinical. Along with all progress above, the CRISPR-mediated gene therapy will ultimately achieve HBV cure.

## 1. Introduction

Hepatitis B virus (HBV) infection remains an important issue of global public health [1]. More than 240 million persons are chronically infected by HBV [2,3]. Chronic HBV infection often results in progressive liver injury and fibrosis, leading to long-term adverse outcomes, including cirrhosis, hepatic failure and hepatocellular carcinoma (HCC) [3,4,5]. It is estimated that up to 40% of patients with chronic hepatitis B (CHB) require timely antiviral treatment to prevent the detrimental outcomes. Although current antiviral therapies, pegylated interferon and nucleos(t)ide analogues (NAs), have dramatically reduced the mortality and morbidity of CHB, neither of them can achieve HBV eradication [6,7]. NA can suppress serum viral load to a level below the detection limit by effectively inhibiting viral polymerase, but rebound viremia frequently occurs after discontinuation of NAs unless loss of hepatitis B surface antigen (HBsAg) is achieved [8,9]. In addition, long-term or even life-long antiviral therapy sometimes causes the problem of poor compliance and the emergence of drug resistance [10]. Therefore, there is a pressing need in development of novel antiviral therapy for HBV cure.

Accumulative evidence has shown that spontaneous or treatment-induced HBsAg loss can only reduce the risk of HCC, but cannot entirely eliminate it. Chronic HBV infection usually leaves permanent scars in the host genome primarily by integration of viral DNA, which may cause dysregulation of cell growth and eventually increase the carcinogenetic risk of infected hepatocytes [11,12].

## 2. HBV Life Cycle and Antiviral Therapy

**Figure 1:**
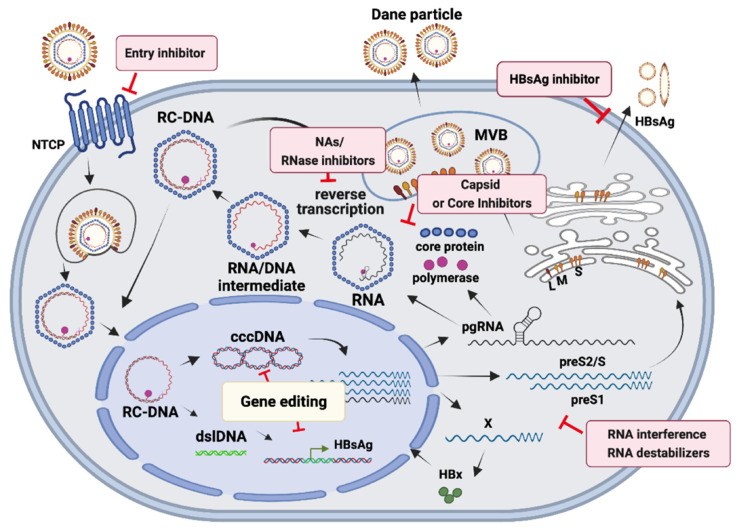
HBV life cycle and current drugs and drugs in development for CHB. The current and candidate antiviral drugs are shown in rectangle box and the targets of inhibitory mechanisms are pointed out by a T-shaped blocking sign.

Understanding the HBV life cycle is required to develop effective antiviral therapy. Here we will only briefly introduce the life cycle and molecular biology of HBV (Figure 1), and more extensive review can be found elsewhere [13,14]. HBV belongs to the *Hepadnaviridae* family. The genome of HBV is partially double-stranded DNA, also called relaxed circular DNA (RC-DNA), and is about 3.2 kb in size, which is so far the smallest genome among animal DNA viruses. It contains four overlapping open reading frames, C, P, S and X, encoding seven viral proteins, including precore (HBeAg), core, polymerase, X (HBx) and the three envelop proteins, L, M and S. HBV infects hepatocytes via binding to its receptor sodium taurocholate cotransporting polypeptide (NTCP) [15]. Upon entry, HBV undergoes endocytosis and uncoating, and its genome RC-DNA is translocated to the nuclei of infected hepatocytes, where the RC-DNA is converted to covalently closed circular DNA (cccDNA), probably through the host DNA repair mechanism [16,17]. HBV cccDNA is the template for viral transcription and replication, from which four viral RNAs are transcribed, including pregenomic RNA/precore transcript, preS1/S2 mRNA, S mRNA and X mRNA. Pregenomic RNA is packaged into the capsid, where it undergoes reverse transcription inside. However, a small portion of RC-DNA is converted to double-strand linear DNA, which either follows the fate of RC-DNA or integrates into the host genome in 1 in 10^5^–10^6^ infected cells [18]. The integrated HBV DNA is defective genome, and fails to generate the pregenomic RNA, so it cannot produce infectious viral genome. Nevertheless, the integrated HBV DNA can transcribe the HBsAg mRNA, so it forms a source for continuous production of HBsAg [19,20]. Besides, integrated HBV DNA can promote the development of HCC, partly through the mechanisms of insertional mutagenesis and transactivating activity [18].

Current antiviral therapy against hepatitis B includes interferon and NAs. Interferon suppresses HBV replication through both direct antiviral and indirect immunomodulating effects, whereas NA does so by inhibiting viral polymerase. However, NA has no effect on the transcription of viral genes from cccDNA and integrated DNA, so it cannot suppress the expression of viral antigens, such as HBsAg (Figure 1). The replication-competent cccDNA forms the major barrier to eradication of HBV infection. In addition, the continuous production of HBsAg from HBV cccDNA and integrated DNA further hampers HBV cure. Therefore, to cure persistent HBV infection, we have to eliminate both cccDNA and integrated HBV DNA [21].

## 3. HBV Cure: Complete Cure versus Functional Cure

**Figure 2:**
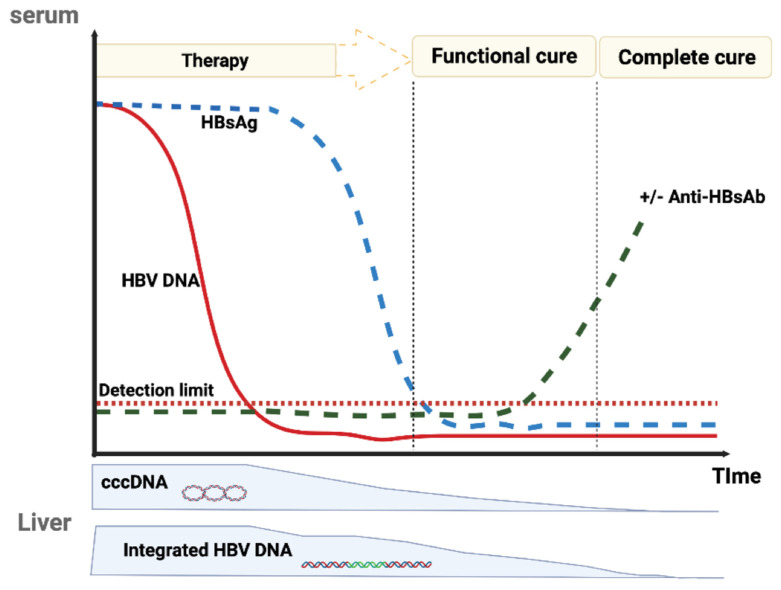
Schematic representation of functional cure and complete cure following antiviral therapy. The HBV DNA, HBsAg, and anti-HBs levels are labeled in red, blue and green dot lines, respectively. The detailed definitions of functional cure and complete cure are referred to the main text.

HBV cure is the ultimate goal of the antiviral therapy, so it must be clearly defined. Two types of HBV cure have been proposed: one is complete cure that needs to purge all the viral genomes from the body of infected persons; the other is functional cure that has been linked to the control of the viral replication and prevention of viral spread after withdrawal of antiviral agents (Figure 2) [22,23]. Complete HBV cure, as the name implies, is defined as undetectability of all the viral proteins and genomes, including intrahepatic cccDNA and integrated HBV DNA. In contrast, functional cure is defined as undetectable HBV DNA in serum and loss of HBsAg with or without the appearance of anti-HBs antibody (HBsAg seroconversion). Currently, it remains extremely difficult to eliminate all the persistent HBV genomes, cccDNA and integrated HBV DNA, to achieve complete cure, so functional cure is a more practical goal to pursue.

## 4. Antiviral Strategies in Development for CHB

Several drugs are under development for treating CHB, including directly interfering the viral replication process and indirectly stimulating the human immune system to control viral spread. New drugs in development target the essential steps of HBV life cycle, including entry inhibitors, capsid or core inhibitors, silencing RNAs, HBsAg release inhibitors, and gene-editing agents (Figure 1). In addition, some drugs modulate human immune system through immunological ways, including therapeutic vaccines, compounds to activate innate immunity, monoclonal antibodies, checkpoint inhibitors, and T cell related therapies. Some of the new therapies are making good progress and have already entered the clinical trials of Phase I or II. The mechanisms and efficacy of the above-mentioned antiviral strategies against HBV have been extensively reviewed in some good articles [24,25], and are beyond the scope of this review. In general, most of the agents that are designed to directly target viral replication steps do not affect the stability and the activity of cccDNA and integrated HBV DNA.

Among these newly developed drugs, the gene-editing approach has attracted wide interest, and represents a unique strategy that can target and inactivate HBV genomes, both integrated HBV DNA and cccDNA, in a sequence-specific manner. It holds great promise in achieving functional cure, or even complete cure. Recently, the cluster regularly interspaced short palindromic repeat (CRISPR)-associated nuclease Cas9 has emerged as a ground-breaking gene-editing tool, and has been applied to destroy HBV genomes in both in vitro and in vivo models (Table 1). Here, we will review the recent progress of CRISPR/Cas in treatment of HBV infection, and point out the challenges and potential solutions for the future application of CRISPR-mediated gene editing for HBV cure.

## 5. CRISPR-Cas9 in Destruction of HBV Genome

**Figure 3:**
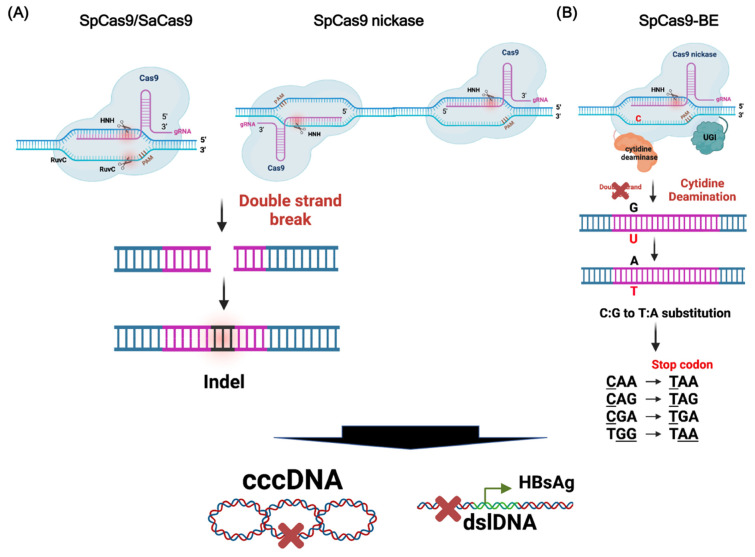
The mechanisms of CRISPR-Cas9 mediated gene therapy against HBV. The cccDNA and integrated HBV DNA are inactivated by (**A**) cleavage with wild-type CRISPR/Cas9 or (**B**) base editing with Cas9-base editor.

The CRISPR/Cas9 system has revealed the revolutionary potential in clinical application because of its extraordinary flexibility and convenience [47,48]. By simply designing the guide RNA (gRNA) complementary to the target DNA sequence, the CRISPR-Cas9 can be redirected to specifically cleave any desired DNA genome, resulting in site-specific DNA double-strand breaks (DSBs). The error-prone nonhomologous end-joining (NHEJ) induced by DSBs often causes frameshift mutations, so the resultant gene deletions or insertions (indels) produce nonfunctional truncated proteins, leading to inactivation of the target DNA genome [49,50]. This feature of the CRISPR/Cas9 gene therapy is particularly suitable to be applied to eradicate or inactivate the persistent HBV genome in CHB patients (Figure 3).

In the past few years, there have been quite a few studies which explored the utility of the CRISPR/Cas9 system as a curative strategy for treatment of CHB. Among them, the prototype *Streptococcus pyogenes* Cas9 (SpCas9) is most extensively used for studying the CRISPR-mediated gene therapy against HBV. These studies took advantage of different in vitro and in vivo HBV expression or infection models. Since each model has its own advantages and limitations for investigating HBV cure, we thus summarized the in vitro and in vivo HBV models used in the studies with CRISPR/Cas9 gene therapy in Table 1. In addition, we will also introduce the novel or engineered Cas9 variants that can be used to overcome the challenges when applying the CRISPR-Cas9-mediated genome editing approach and the in vivo delivery systems to demonstrate the therapeutic effect of CRISPR/Cas9, which are summarized in Tables 2 and 3, respectively.

### 5.1. In Vitro and In Vivo HBV Models for Gene Editing of HBV Genomes by CRISPR-Cas9

There have been a number of studies, including ours, to explore the potential of the CRISPR/Cas9 system in specific disruption of HBV genomes. These works utilized different in vitro and in vivo HBV replication models to demonstrate the effect of the CRISPR/Cas9 system in inactivation or destruction of HBV genomes, and are summarized in Table 1.

There are several in vitro HBV models that are used to determine the effect of CRISPR-Cas9, including transfection of HBV-expressing plasmid, cell lines harboring the integrated HBV genome, and in vitro HBV infection system. The first one is a convenient and flexible method to establish the in vitro HBV model by simply transfection of HBV-expressing plasmid of interest into hepatoma cell lines. A number of studies have used this approach to examine the efficacy and specificity of gRNAs [26,27,28,29,30,31,32,33,34,35,36,37,38]. The drawbacks of this approach are the transient expression of HBV genes and no production of cccDNA. The second in vitro HBV model is the cell lines harboring the integrated HBV genome. In this model, cells are stably transduced with at least a full-length HBV genome, from which HBV genes are constitutively expressed. Although no cccDNA is produced in this model, it is usually utilized to demonstrate the effect of the CRISPR-mediated gene editing on mutagenesis or removal of the integrated HBV genome [27,28,30,36,38,39,40,41,42]. The HBV infection model counts on HepaRG cells or cells stably expressing NTCP that are susceptible to HBV infection. The establishment of this model is time- and labor-intensive and requires the use of high-titer infectious HBV virions. However, only this model can generate cccDNA, whose mutagenesis and destruction by CRISPR-mediated genome editing had been shown in several studies, supporting its potential for HBV cure [28,30,31,32,38,39,40,43,44].

Among the in vivo HBV models, hydrodynamic injection (HDI) with HBV-expressing plasmid or precursor cccDNA (precccDNA) with Cre-expressing plasmid is commonly used. Viral genes are only expressed in a small portion of hepatocytes (<10%) and there is no viral spread or transmission, but HBV persistence can last several weeks or even months. This model is advantageous for its convenience to establish, and ease to demonstrate the effect of CRISPR-mediated genome editing against HBV [26,27,29,30,32,35,36,38,41]. HBV-transgenic mice are also often used to examine the efficacy of CRISPR-Cas9-mediated gene therapy for hepatitis B. Although there is no cccDNA production nor viral spread in this model, several studies had shown the therapeutic effect of CRISPR-Cas9 on integrated HBV genomes [32,34,37,41]. Humanized hepatocyte chimeric mouse models are considered an ideal HBV infection model that can faithfully reflect human HBV infection. Although expensive, highly technically dependent, and immune-deficient, these human hepatocyte chimeric mouse models with HBV infection can generate cccDNA, so they are indeed the gold standard to examine the antiviral effect of the CRISPR-mediated gene editing on HBV infection. Two studies took the AAV delivery system with split SpCas9 or SaCas9 to determine the antiviral activity against HBV [45,46]. Of note, although the results showed the successful gene editing of persistent HBV infection, the efficacy was only modest, suggesting that there remains room for improvement of in vivo delivery efficiency of CRISPR-Cas9.

### 5.2. Challenges for Eradication of HBV by CRISPR/Cas9

Although the CRISPR/Cas9 system provides strong evidence to support its utility in specific destruction or inactivation of HBV genome, there remain significant challenges that need to be cautiously addressed before its clinical application. Firstly, the cleavage of the integrated HBV DNA by the prototype Cas9 can cause host DNA DSBs with resultant large deletions and complex rearrangements of host genome, which increase the risk of genome instability and carcinogenesis [51]. Secondly, the off-target effect of CRISPR/Cas9 needs to be carefully evaluated. Thirdly, HBV is featured by the high sequence heterogeneity within and between genotypes, which poses serious difficulty in finding effective gRNAs that can target conserved HBV sequences across different genotypes. Last, the large size of CRISPR/Cas9 gene renders in vivo delivery extremely challenging for therapeutic applications. The large prototype SpCas9 is difficult to fit into the commonly used and clinically approved delivery vector, the adeno-associated viral (AAV) vector. In addition, the delivery specificity for particular specific tissue and cell types and immunogenicity of the in vivo delivery system should be resolved.

## 6. Tackling the Challenges of CRISPR-Mediated Gene Therapy for Hepatitis B

To tackle the challenges facing the CRISPR-mediated gene therapy against HBV, several strategies have been developed, including novel Cas9 variants and delivery approaches, which are summarized in Table 2 and Table 3, respectively.

### 6.1. Cas9-BE for Permanent Inactivation of HBV Genome without Inducing DSBs of Host Genome

The CRISPR/Cas9 system not only destructs HBV cccDNA, but also cleaves the integrated HBV DNA. The latter leads to DSBs of the host genome and may induce complex genome rearrangement and large genes deletions. The cytosine base editor (CBE) is a newly developed CRISPR-derived base editing tool that allows for precise conversion from a C-G base pair to a T-A base pair in the target genomic locus without inducing DSBs [52]. Thus, CBEs, named BEs hereafter, can be used to introduce premature stop codon into specific genome for permanent inactivation of genes of interest without DNA DSBs, so it is particularly suitable for inactivating integrated HBV DNA.

The BEs are initially designed by tethering the APOBEC deaminase to “dead” SpCas9 (dCas9), which is catalytically inactivated by introduction of both mutations D10A/H840A. To further improve the efficacy of BE, the uracil glycosylase inhibitor (UGI) is fused to Cas9 nickase (Cas9n) instead of dCas9, resulting in BE3, a single protein consisting of tripartite components, including APOBEC1, Cas9n and UGI [50]. The further evolved BE called BE4Gam is developed for increasing the C-G to T-A efficacy and reducing the indel formation by fusing the codon-optimized BE3 with Gam from bacteriophage Mu [53,54,55]. Our previous study utilized CRISPR/Cas9-mediated BEs to successfully introduce nonsense mutations to HBV cccDNA and integrated DNA, which permanently silenced HBV polymerase and surface genes to reduce the expression of HBV DNA and HBsAg. Our study provides the first proof-of-concept that CRISPR/Cas9-mediated base editing can permanently silence HBV gnomes without creating DSBs of host genome (Figure 3) [21].

### 6.2. Engineered Cas9 for Improving the Target Specificity

The off-target effect is a major concern for the clinical application of Cas9. Several strategies have been utilized to generate an array of novel Cas9 variants with greatly enhanced on-target specificity, including Cas9 nickase (Cas9n), catalytically inactive Cas9 fused with FokI nuclease (fCas9), rationally engineered Cas9 with high fidelity (Cas9-HF1, HiFi-Cas9), or enhanced specificity (eSpCas9), and evolved Cas9 with broad PAM and high specificity (xCas9) [56,57,58,59,60]. Cas9n with a pair of appropriately offset gRNAs induces DSBs of the target sequence through paired nicking, which can reduce the off-target activity by 50- to 1500-fold [61]. The Cas9n-mediated gene-editing strategy has been applied to disruption of HBV genome with improved target specificity [33,36,61]. Besides, fCas9 is produced by fusion of the dimerization-dependent FokI nuclease domain with the catalytically inactive dCas9, which can greatly enhance the Cas9 specificity because similar to Cas9n, it requires a pair of gRNAs that recruit two fCas9 to target sites together for DNA cleavage activity [60]. Moreover, wild-type SpCas9 is engineered by creating point mutations to generate high-fidelity Cas9 variants, including SpCas9-HF1, HiFi-Cas9, and eSpCas9. SpCas9-HF1 was derived from SpCas9 with the quadruple substitutions (N497A/R661A/Q695A/Q926A), HiFi-Cas9 from SpCas9 with single substitution R691A, and eSpCas9 from SpCas9 with triple substitutions (K848A/K1003A/R1060A). xCas9 is a SpCas9 variant with broad PAM compatibility and high DNA specificity that was generated through phage-assisted continuous evolution [57]. The detailed comparison among these Cas9 variants has been reviewed and can be found elsewhere [62]. All these newly developed Cas9 variants with enhanced on-target specificity are expected to improve the safety in CRISPR-mediated gene therapy for CHB.

### 6.3. Smaller Cas9 for Efficient in Vivo Delivery by AAV Vectors

The large cargo size of SpCas9, 1368 amino-acid residues or around 4.1 kilobase pairs, poses a large challenge for its in vivo delivery by the AAV vector, which has limited cargo capacity. In this regard, Cas9s with smaller size will have higher efficiency for in vivo AAV delivery. *Staphylococcus aureus* Cas9 (SaCas9) has been discovered and featured by its small size with only 1082 amino-acid residues or around 3.2 kb, so it has been utilized for delivery by AAV vectors [63]. SaCas9 delivered by AAVs have been examined for its effects on treatment of HBV in vitro and in vivo [37,38,43,45]. Another Cas9 derived from *Campylobacter jejuni* (CjCas9) also has small-sized gene with 2955 nucleotides encoding a 984 amino-acids protein [64]. Of note, SaCas9 requires the 5′-NNGRRT-3′ PAM, whereas CjCas9 requires the 5′-NNNNACAC-3′ PAM. It is more difficult for these two Cas9s to find candidate gRNA-compatible sequences because of their requirement of longer PAMs.

### 6.4. Cas9s with Less Restricted PAMs for Broadening the gRNA Choices

Currently, at least nine genotypes of HBV, from genotype A to genotype I, have been identified [65,66]. Given the high heterogeneity of HBV sequences, it is difficult to find effective gRNAs that can target sequences conserved across a wide range of genotypes or even pan-genotypes of HBV. This issue is particularly critical when Cas9-BE is used because it has a narrower pool of candidate gRNAs than the prototype SpCas9 [21]. One strategy to broaden the candidate gRNA pool for choice is to loosen the restriction of PAM. SpCas9 is thus engineered to generate several evolved SpCas9 variants with less restriction of PAM. For example, VQR-, VRER-, EQR- SpCas9 variants recognize altered PAMs, NGAN, NGCG, and NGAG, respectively [67]. In our previous study, we took advantage of VQR-, VRER-, and EQR- SpCas9-BE for inactivating HBV genome, but found that they had significantly lower base-editing efficiency compared to wild-type SpCas9-BE [67]. Recently, SpG and SpRY are reported to have minimal requirement of PAM, targeting NGN PAM and NRN/NYN PAM, respectively [68]. In addition, a novel *Streptococcus canis* Cas9 (ScCas9) recognize a less restricted 5′-NNG’-3′ PAM [69]. These PAMless Cas9s will certainly facilitate the discovery of effective and conserved gRNAs for CRISPR/Cas9-mediated treatment of hepatitis B.

### 6.5. Delivery of CRISPR/Cas

It has been challenging to deliver CRISPR/Cas9 to the target organs or tissues in vivo because of its large cargo size. Viral and non-viral vectors are two major methods for the in vivo CRISPR/Cas9 delivery. Here, we will only briefly introduce a number of CRISPR/Cas9-delivering vectors that have been utilized to demonstrate the efficacy for treating HBV infection (Table 3).

The AAV vector is currently approved for gene therapy of several genetic diseases in clinical [70]. It is advantageous for its low pathogenicity and immunogenicity, wide range of cell tropism, and long-term expression [71]. However, several studies have shown that high titer of AAV-SaCas9/gRNAs (>10^11^ viral genomes per mouse) is usually required for effective and observable genome editing [37,38,43,45]. In addition, the cargo capacity of the AAV vector is limited, so only small Cas9s, such as SaCas9, can fit into the AAVs for treatment of HBV infection as described above [63]. Therefore, to overcome the obstacle of cargo capacity, an alternative strategy utilizes two Cas9-fragment system [46] or split-intein Cas9 system, in which a full functional Cas9 protein is split into two non-functional fragments. Upon delivery to the target cells, the two fragments of Cas9 is reconstituted to a fully functional protein through either recombination [46] or the intein-mediated trans-splicing mechanism [72], but this approach usually requires high viral titers because co-delivery of dual AAVs into the same cells is less efficient. Additionally, AAVs also bear the potential risk of integration of viral DNA genome into the host genome, causing genetic mutations and carcinogenesis, induction of adverse immune responses, and constitutive expression of Cas9, which raises unignorable safety concerns. Moreover, the long-term expression of Cas9 from the AAV vector tend to increase the risk of off-target effect.

Adenoviral vector is another viral vector that shows the potential for in vivo delivery of the CRISPR-Cas9 system. It has larger cargo capacity than AAV vectors, high transduction capacity and a wide range of cell tropism. A recent study demonstrated that high-capacity adenoviral vectors could be utilized for delivery of multiplex gRNAs with CRISPR-Cas9 nuclease in a single vector to disrupt cccDNAs in HBV-infected cells [44]. Nevertheless, adenoviral vectors also have the safety concerns for its risk of integration into host genome and elicitation of adverse inflammatory response. Moreover, repeated delivery by adenoviral vectors will encounter the problem of declining efficacy because repeated exposure to adenoviral vectors tends to induce the vector-targeting antibody response [73].

Non-viral vectors are synthetic materials, such as lipid or polymers, and advantageous for their flexible cargo capacity and high safety profile in clinical applications. Extensive review on this topic can be found elsewhere [74,75]. Here we will only briefly introduce non-viral vectors for delivery of CRISPR/Cas9 in treatment of HBV. HDI with Cas9-gRNA-expressing plasmids are commonly used in demonstration of its in vivo genome-editing efficacy against HBV in mouse models, but not in clinical setting. Although convenient, it has low efficiency of in vivo transfection and transient Cas9 gene expression. Lipid nanoparticles (LNPs) are commonly used in delivery of ribonucleoprotein (RNP) or DNA/RNA, and advantageous for its low toxicity and immunogenicity when applied in vivo. Suzuki et al. generated LNP for the delivery of CRISPR/Cas9 RNPs, and exhibited significant editing of HBV genome in a cell-based system [76]. Moreover, Wei et al. successfully demonstrated the in vivo delivery CRISPR/Cas9 ribonucleoprotein (RNP) and editing of several tissues including muscles, brain, liver, and lungs [77]. In addition, Zhang et al. developed functionalized TT derivatives 5 (FTT5) lipid-like nanomaterials (LLNs) for long mRNA delivery, and showed it could achieve more than 50% gene editing in a mouse model by in vivo delivery of Cas9-base editors mRNAs [78]. Interestingly, LLN-packaged mRNA and gRNA has been shown to be effective in vivo gene editing of HBV DNA in an HDI-based HBV persistence mouse model [35]. For therapeutic applications, safe and efficient delivery of CRISPR/Cas9 components into the nuclei of HBV-infected hepatocytes is critical for in vivo gene editing of HBV genome.

**Table 3:** Viral and nonviral delivery vectors for studying the effect of CRISPR-Cas9-mediated gene editing on HBV genome.

Category	Advantages	Disadvantages	Reference
**Delivery of Cas9 by viral vector**			
AAV	It has been approved for clinical use in genetic diseases. It has low pathogenicity and immunogenicity, wide range of cell tropism and long-term gene expression.	It has limitation of cargo capacity. The risk of DNA integration into host genome. Long-term Cas9 gene expression may lead to a higher risk of off-target effect.	[37,38,43,45,46]
Adenovirus	It has larger cargo capacity than AAV vectors. It has high transduction efficiency and a wide range of cell tropism.	It has a risk of integration of viral DNA into host genome. It induces the inflammatory response.	[44]
**Delivery of Cas9 by nonviral vector**			
HDI with Cas9-expressing plasmid	It is convenient in mouse models.	It is not practical in clinical setting. The in vivo delivery efficacy is low.	[26,27,29,30,32,34,35,36,41]
RNP or mRNA/LLN (lipid-like nanoparticles)	It is convenient and efficient for delivery of Cas9. It has lower cytotoxicity and immunogenicity, and no risk of DNA integration to host genome. Its transient expression of Cas9 results in lower off-target risk. It has larger cargo capacity than the AAV vectors.	The cost of production is higher than that of viral vectors.	[35,76]

## 7. Summary and Future Perspectives

Decades of research on HBV has unveiled the mysterious HBV life cycle and pathogenesis, and identified the intrahepatic cccDNA and integrated DNA as the formidable obstacles to HBV cure. Several novel strategies are in development, aiming to achieve functional or even complete cure for CHB. The enthusiastic pursuit of HBV cure further gains momentum from the recent success of direct antiviral agents in HCV cure. Standing out of the potentially curative approaches, the CRISPR gene-editing technology is uniquely featured by its site-specific cleavage, opening a whole-new opportunity to directly target persistent HBV genomes for achieving HBV cure. Of note, the safety issue of any new antiviral therapy aiming to cure CHB requires special attention given that current long-term antiviral therapy with NA is safe and effective [14,22].

Although promising, the CRISPR-Cas9 system faces several daunting challenges as stated above, which need to be overcome before its clinical use. A number of strategies or novel tools have also been proposed or discussed above in order to resolve these problems. The CRISPR-Cas9-derived base editor can be utilized to permanently inactivate HBV genome for suppression of HBsAg production and HBV replication without DSBs of the host genome, providing superior safety compared to prototype SpCas9. Additionally, the application of Cas9 with high-fidelity and broad PAM compatibility for treatment of hepatitis B can reduce the off-target risk and broaden the gRNA pools targeting conserved HBV DNA sequences across different genotypes of HBV. Moreover, the recent advances in CRISPR-Cas9 delivery strategies with high efficacy and safety has further facilitated its clinical application. The inspiring success of LNP-mRNA-mediated delivery of CRISPR-Cas9 to liver in a very recent clinical trial for treating transthyretin amyloidosis lends strong support to develop future gene therapy against CHB [79].

All progress above will eventually lead to the realization of CRISPR-mediated gene therapy in treatment of hepatitis B. It is foreseeable that to ultimately achieve HBV cure, the CRISPR-mediated gene therapy needs to combine with NA therapy which can completely stop HBV replication. Although HBV functional cure is a more practical goal, the pursuit of complete HBV cure may be still necessary. Recent study has shown that the integrated HBV DNA can exhibit transactivating activity on the adjacent oncogenes, such as telomerase, to promote the development of HCC, so the risk of HCC remains unneglectable even after HBsAg loss or functional cure. Therefore, complete cure with the CRISPR-mediated gene therapy to purge all the HBV genome will be the final pursuit in the long journey to conquer hepatitis B.

## Figures and Tables

**Table 1:** In vitro and in vivo HBV models for studying the effects of CRISPR-Cas9-mediatd gene editing.

Category	Features and Advantages/Disadvantages	Reference
**In vitro model**		
Transfection of cell lines with HBV-expressing plasmid	It is convenient and flexible to establish by transfection with HBV of genotypes of interest. It can be used to demonstrate the gRNA specificity and efficacy in cleavage of HBV genome. Viral genes are transiently expressed. No cccDNA is produced.	[26,27,28,29,30,31,32,33,34,35,36,37,38]
Cell lines harboring the integrated HBV genome	A full-length HBV genome is integrated in the human genome. It stably expresses all the viral genes, but produces no or only very few cccDNAs. It has been used to demonstrate the mutagenesis or removal of integrated HBV DNA by CRISPR/Cas9.	[27,28,30,36,38,39,40,41,42]
HBV infection system	It is time- and labor-intensive to establish, and requires skillful techniques. It can convincingly generate measurable cccDNAs. It is a well-established model that is used to show the reduction or mutagenesis of cccDNA by CRISPR/Cas9.	[28,30,31,32,38,39,40,43,44]
**In vivo model**		
HDI with HBV-expressing plasmid or precccDNA	It is easy to establish, but only <10% of hepatocytes are transfected with HBV-expressing plasmid. The viral genes are expressed in a low level, but no cccDNA is generated. There is no viral spread and transmission among hepatocytes.	[26,27,29,30,32,35,36,38,41]
HBV-transgenic mice	Every hepatocyte contains the integrated HBV genome. There is no HBV infection or spread. HBV genes are tolerated by the host immune system.	[32,34,37,41]
Human hepatocyte chimeric mice with HBV infection	It is a true HBV infection model, in which human hepatocytes are susceptible to HBV infection. The infected human hepatocytes harbor cccDNA. It is expensive and time- and labor-intensive to establish, and requires skillful techniques. The mice are immune-deficient, and the turn-over rate of human hepatocytes is high.	[45,46]

**Table 2:** Engineered or new Cas9 variants that have been applied for HBV treatment.

Category	Advantages	Disadvantages	Reference
SpCas9-BE (2016)	It inactivates HBV genomes by introduction of premature stop codons without inducing DSBs. It avoids DSBs in the integrated HBV DNA of host genome.	It has a smaller pool of candidate protospacer sequences due to the requirements for target base-editing sites and PAM. It has larger gene size.	[21]
SpCas9 nickase (2013)	It enhances the specificity of target cleavage by producing two nicks on two. opposite strands of DNA with a pair of gRNAs.	It has larger gene size. Two gRNAs are required to cleave one site.	[28,33,36]
SaCas9 (2015)	It has smaller Cas9 size, so it fits into the. AAV vectors.	It has a smaller pool of candidate gRNAs. due to the requirement of the longer 5′-NNGRRT-3′ PAM.	[37,38,43,45]
Cas9 with less restriction of PAM (2015)	It loosens the restriction of PAM. It can broaden the pools of candidate gRNAs targeting the conserved HBV sequences, particularly for Cas9-BE.	The efficacy of Cas9 variants may be lower than wild-type Cas9.	[21]
